# Three cases of recalcitrant *Paecilomyces* keratitis in Southern California within a short period

**DOI:** 10.1186/s12348-023-00380-z

**Published:** 2024-01-04

**Authors:** Christine K. Kim, Joseph T. Mekhail, David M. Morcos, Christopher D. Yang, Sanjay R. Kedhar, Cinthia Kim, Maria Del Valle Estopinal, Olivia L. Lee

**Affiliations:** 1 Gavin Herbert Eye Institute, University of California, Irvine School of Medicine, 850 Health Sciences Rd, Irvine, CA 92617 USA; 2 Department of Pathology, University of California Irvine, Irvine, CA USA

**Keywords:** Fungal keratitis, *Paecilomyces*, Posaconazole

## Abstract

**Background:**

The aim of this report is to describe the risk factors, clinical course, and characteristics of three cases of *Paecilomyces* keratitis presenting concurrently within three months in the same location. We used in vivo confocal microscopy and histopathology to corroborate our clinical findings.

**Observations:**

Three eyes of three elderly patients with culture-proven *Paecilomyces* keratitis were included in this series. These patients resided within a 15-mile radius and presented to a tertiary care eye institute in Southern California between February and April 2022. All three eyes experienced a prolonged, recalcitrant course with recurrence of keratitis in donor corneal tissue despite antifungal therapy and multiple therapeutic penetrating keratoplasties. In vivo confocal microscopy, histopathology, and microbiologic findings corroborated the diagnosis of fungal keratitis with *Paecilomyces*. With surgical intervention and extensive medical therapy, all three cases resolved after the addition of oral Posaconazole.

**Conclusions:**

*Paecilomyces* is a rare cause of infectious keratitis. Herein we report three similar cases in elderly patients. All had prolonged, recalcitrant infections that required multiple treatment modalities. Our cases, which were supported by in vivo confocal microscopy and histopathology, highlight the importance of timely and aggressive therapy to prevent recurrence.

## Introduction

*Paecilomyces* is a ubiquitous filamentous fungus commonly found in soil and air [1]. *Paecilomyces* rarely causes disease in humans; however, there have been increasing cases of infection in recent years [2]. Infection gives rise to a variety of clinical presentations, including but not limited to, keratitis, endophthalmitis, and cutaneous infections [2]. Established risk factors for infectious keratitis include contact lens wear [2], trauma to ocular surface [3], and chronic topical corticosteroid use [3]. The outcomes of fungal keratitis vary, and treatment is usually initiated with topical antifungal drugs [4].

The first line of treatment is topical natamycin [4]. The Mycotic Ulcer Treatment Trial (MUTT I) demonstrated the efficacy of natamycin over voriconazole in preventing corneal perforation and penetrating keratoplasty in smear or culture-proven fungal keratitis [4]. Intracameral amphotericin B has also been reported as an effective treatment in deep fungal corneal ulcers [5]. Despite consistent topical and intracameral antifungal therapy, treatment may fail. Oral posaconazole has demonstrated efficacy in treating resistant cases [6]. Even with these available treatments, infection with *Paecilomyces* has been associated with poor outcomes: 60% of the eyes required keratoplasty and 19%–35% resulted in enucleation of the eye [7, 8]. In this case series, we report the clinical progression of three patients with *Paecilomyces* keratitis, using in vivo confocal microscopy and histopathology to substantiate our clinical and microbiologic findings.

### Patients and methods

This retrospective case series examined the risk factors, clinical course, and characteristics of three patients with *Paecilomyces* keratitis presenting concurrently within three months in the same location. Data collected retrospectively included patient demographics, preexisting ocular conditions, exam findings, presenting symptoms, and treatment course (Table 1).

**Table 1:** Patient characteristics and medications

Patient Characteristics	Case 1	Case 2	Case 3
Age (Years)	72	87	77
Gender	Female	Female	Male
Ocular History	Contact lens-related corneal ulcer OD	Recurrent herpetic anterior uveitis OS; bilateral primary open-angle glaucoma	PKP for corneal ulcer complicated by descemetocele formation OD; bilateral glaucoma
Contact Lens Wear	Yes	No	No
Baseline VA (OD; OS)	N/A	20/50; 20/60	CF at 2 ft; CF at 3 ft
Presenting VA (OD; OS)	20/600; 20/30	20/30; 20/500	HM; CF at 3 ft
Presenting Symptoms	Severe eye pain and worsening vision	Severe eye pain and worsening vision	Persistent epithelial defect and occasional eye pain
Topical Medications	Vancomycin 25 mg/mL Tobramycin 15 mg/mL Moxifloxacin Prednisolone Acetate 1% Trimethoprim / Polymyxin Natamycin	Loteprednol Travoprost Brimonidine / Timolol Besifloxacin Erythromycin Natamycin Trimethoprim / Polymyxin Moxifloxacin Neomycin / Polymyxin b / Dexamethasone	Cenegermin Timolol Brimonidine Latanoprost Moxifloxacin Loteprednol Natamycin Prednisolone Acetate 1%
Intraocular Injections	Voriconazole 100mcg/0.1 cc Amphotericin B 5mcg/0.1 ml	Voriconazole 100mcg/0.1 cc Amphotericin B 5mcg/0.1 ml	None
Oral Medications	Voriconazole 200 mg BID Doxycycline 100 mg BID	Voriconazole 200 mg BID Doxycycline 100 mg BID Valaciclovir 1000 mg QD	Voriconazole 200 mg BID Doxycycline 100 mg BID
Final Medication Regimen Before Resolution	Posaconazole 300 mg BID for 2 weeks, then QD for 2 weeks	Posaconazole 300 mg BID for 2 weeks, then QD for 2 weeks	Posaconazole 300 mg BID for 3 weeks

*Abbreviations*: *OD* Oculus dexter (Right eye), *OS* Oculussinister (Left eye), *PKP* Penetrating keratoplasty, *N/A* Not available, *CF* Counting fingers, *HM* Hand motion, *BID* Bis in die (Twice a Day), *QD* Quaque die (Once a Day)

### Findings

#### Case 1

A 72-year-old female with a history of contact lens-related corneal ulcer in the right eye was referred to our department in February 2022 for acute corneal perforation. The patient reported right eye pain that began two months prior to presentation. On examination, best corrected visual acuity was 20/600 in the right eye. Slit lamp examination revealed a 5 mm central perforated corneal ulcer with clear peripheral cornea. The patient underwent a therapeutic penetrating keratoplasty (PKP) on the same day. The host cornea was sent for histopathologic evaluation and microbiology studies including bacterial and fungal cultures.

In the immediate post-operative period following tectonic PKP (before microbiologic and histopathologic results were available), the patient was treated with fortified vancomycin, fortified tobramycin, moxifloxacin, and prednisolone. After two weeks, the epithelial defect resolved, and the cornea appeared clear. However, the following month, the patient presented with a small endothelial plaque superiorly on the donor cornea with hypopyon that spread to the host cornea site, prompting treatment with moxifloxacin, trimethroprim and polymyxin B, natamycin, voriconazole, and doxycycline. At this time, best corrected visual acuity was hand motion.

**Fig. 1:**
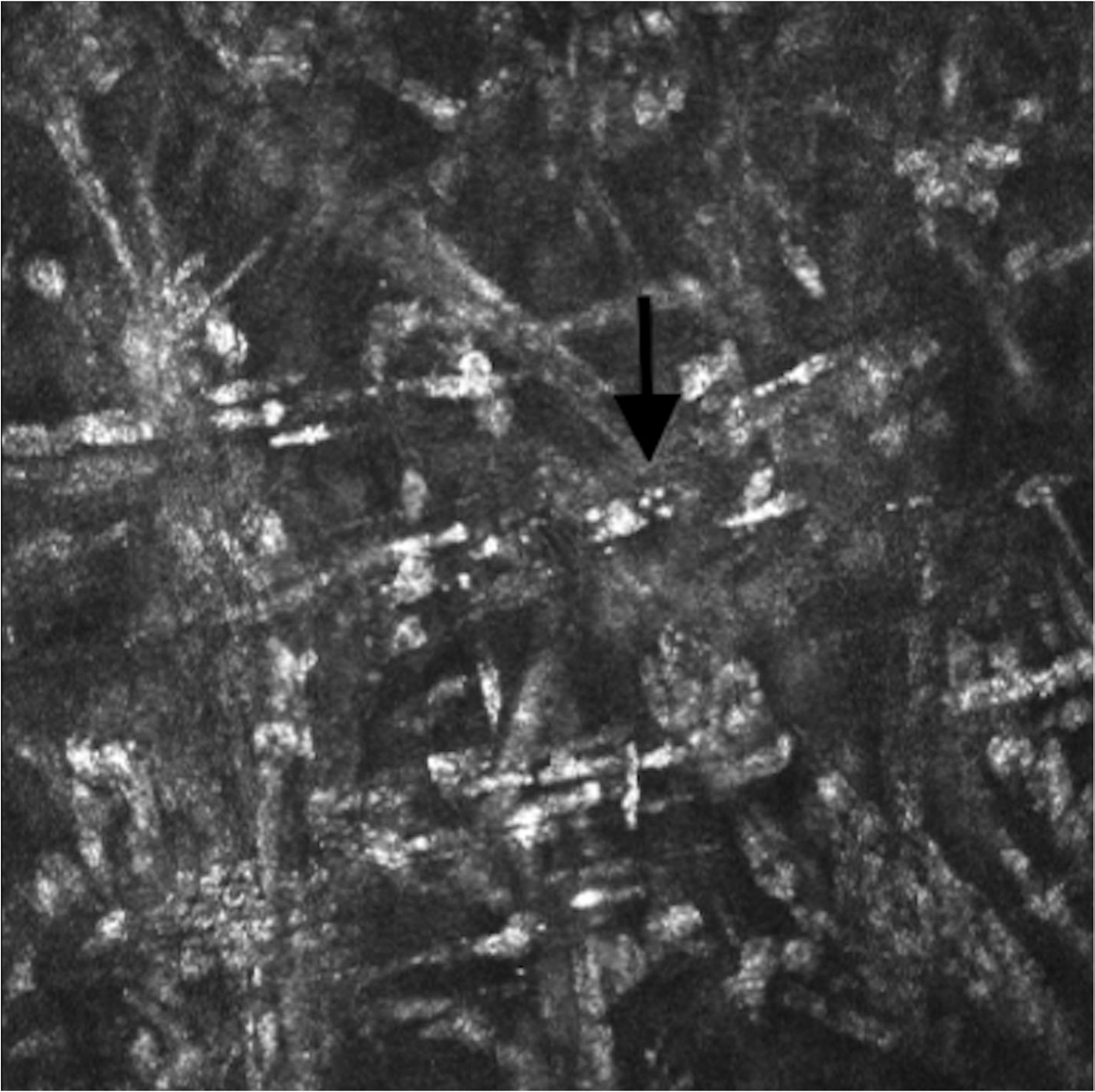
In vivo confocal microscopy showing small hyperreflective round structures, indicative of spores (arrow)

Corneal scrapings were taken for gram staining and culture examination using the standard microbiological culture techniques. Fungal isolates were identified through their morphology, which were speciated as *Paecilomyces*. Additionally, in vivo confocal microscopy (IVCM) of the donor cornea exhibited signs of spores (Fig. 1). The patient underwent a second therapeutic PKP with anterior chamber washout and intracameral antibiotics and antifungals.

The histopathologic assessment of the penetrating graft confirmed acute stromal necrotizing inflammation associated with fungal elements which were percolating Descemet membrane.

**Fig. 2:**
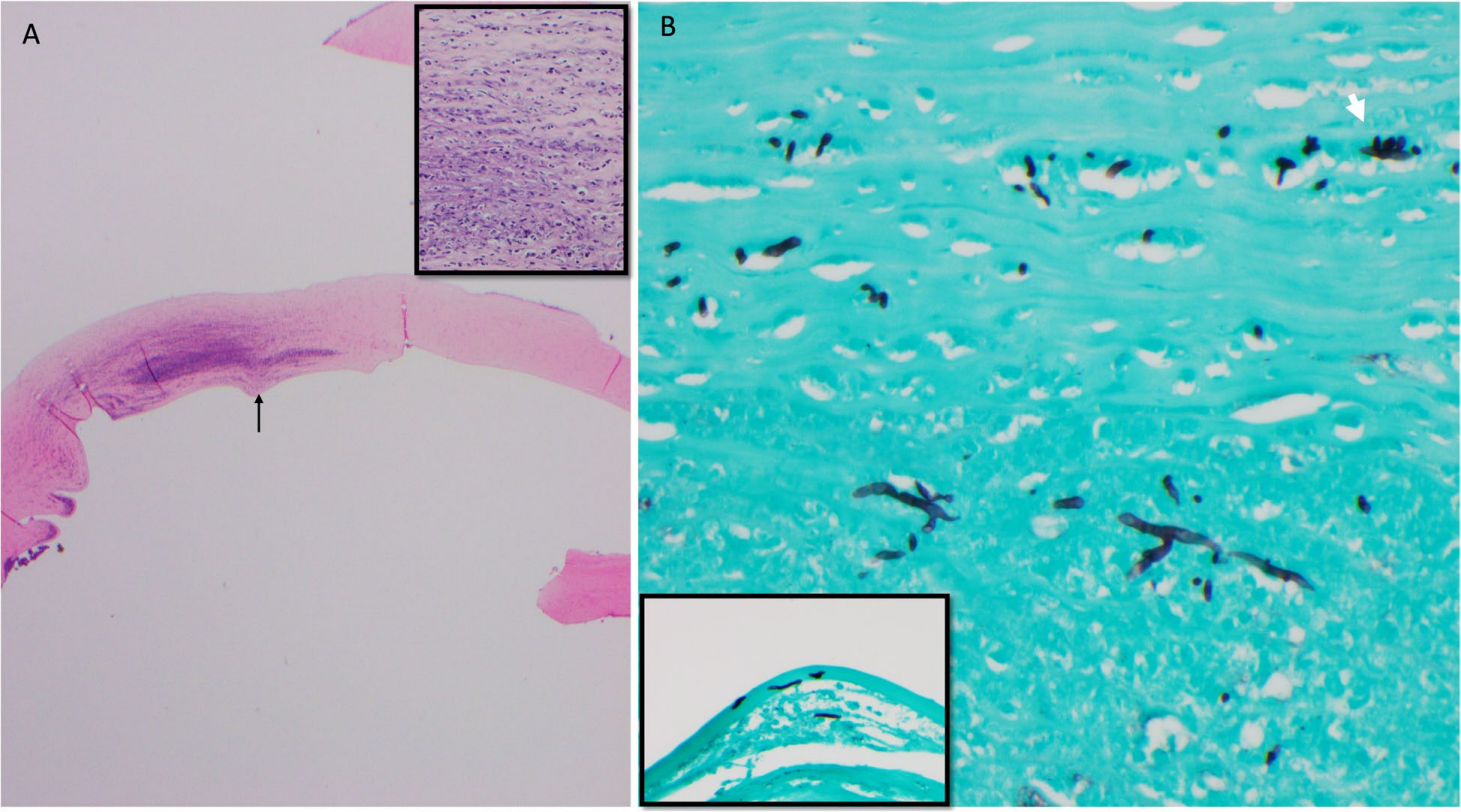
A Penetrating corneal graft depicting dense stromal abscess and Descemet membrane folds (black arrow). H&E, original magnification × 20. Inset: corneal stroma with collections of neutrophils mixed with cellular debris. H&E, original magnification × 400. B. Grocott methenamine silver stain highlighting branching septate hyphae and chlamydospores (white arrow) in the abscess. GMS original magnification × 400. Inset: fungal elements percolating Descemet membrane. GMS × 400

The epithelium and Bowman layer were focally absent and associated with extensive stromal thinning, necrosis, and a dense infiltrate of neutrophils, forming microabscesses. The Descemet membrane was focally attached and revealed folds and loss of endothelial cells. Histochemical studies, periodic acid-Schiff (PAS) and Grocott’s methenamine silver (GMS) stains highlighted groups of hyphae- and yeast-like fungal elements infiltrating the posterior lamellae (Fig. 2). The concurrent microbiology cultures from the excised cornea showed growth of mold, specifically *Paecilomyces* species.

**Fig. 3:**
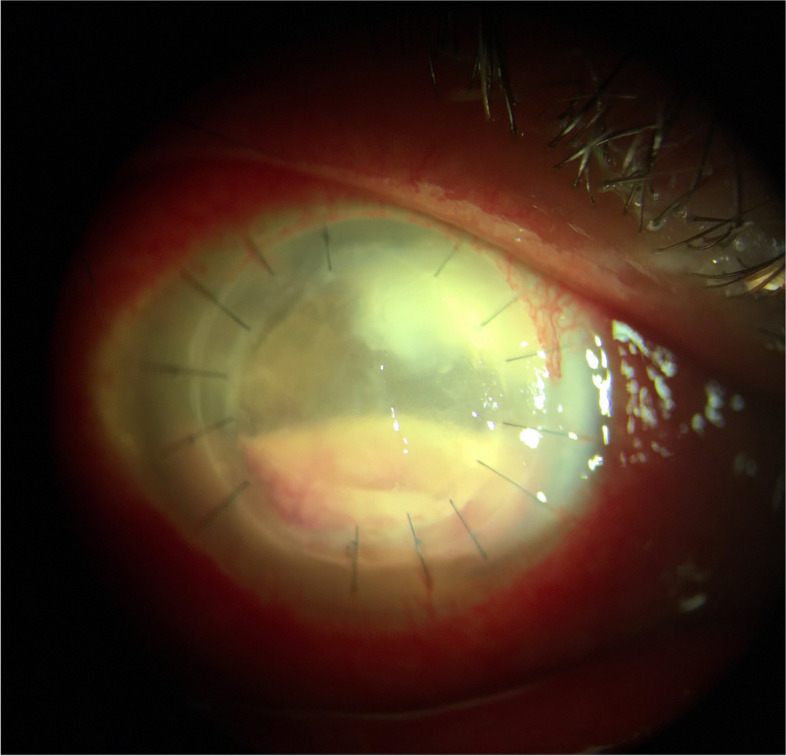
Endothelial plaque and hypopyon prior to second therapeutic PKP

The patient continued to take antifungals and antibiotics including voriconazole, natamycin, moxifloxacin, and trimethoprim/polymixin, with no evidence of recurrence for eight weeks. However, an additional eight weeks after, the patient reported new-onset right eye pain despite the use of these antifungals and antibiotics. On examination, there was a deep stromal infiltrate in the graft host junction at 11 o’clock, accompanied by a 1.1 mm hypopyon and white cataract (Fig. 3). Visual acuity remained hand motion. Aggressive treatment for fungal keratitis was employed, which involved continuing oral voriconazole and increasing the frequency of topical voriconazole and natamycin. In addition, intrastromal amphotericin B and voriconazole injections were given on three occasions without resolution of the infiltrate.

**Fig. 4:**
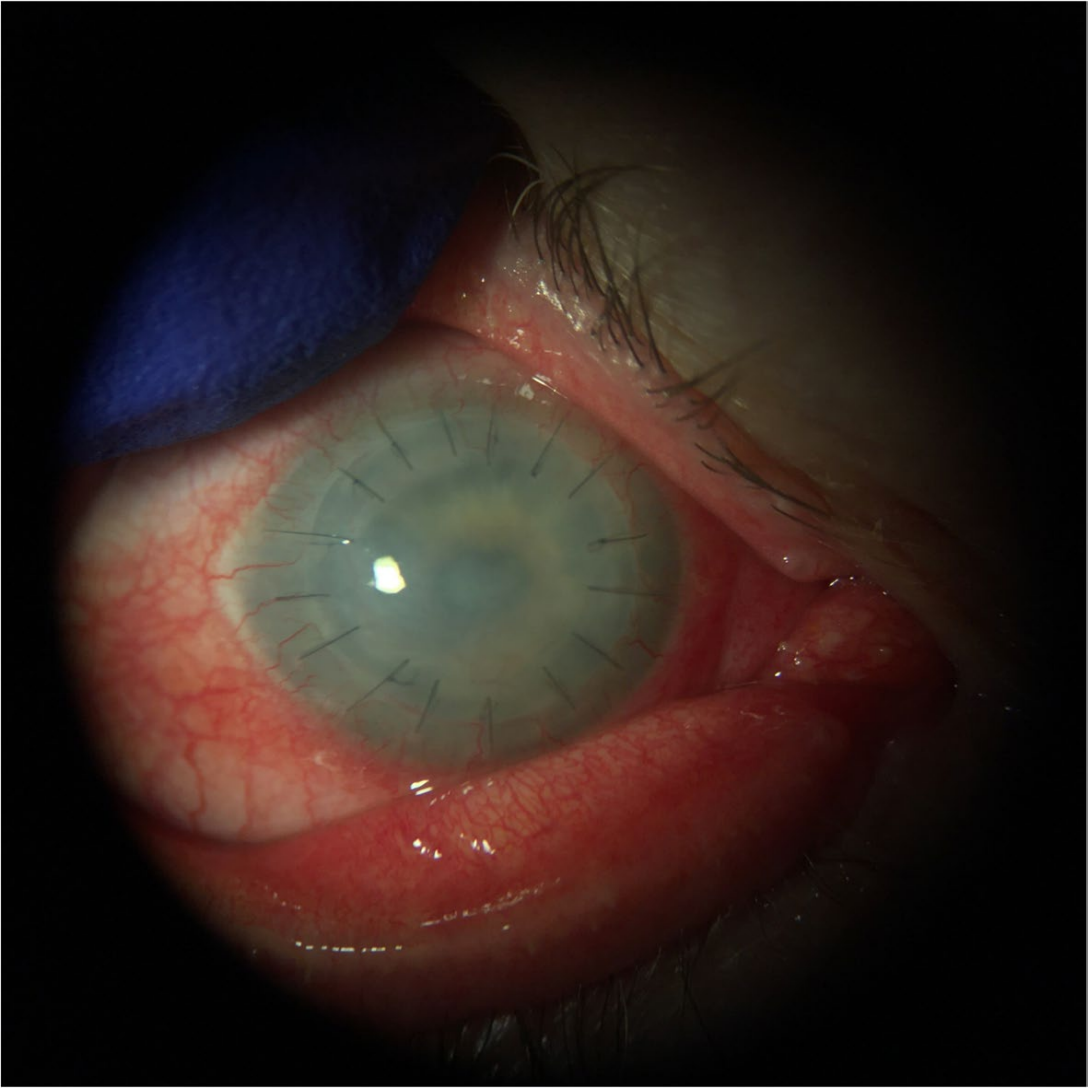
Resolution of infectious keratitis without recurrence following treatment with systemic Posaconazole for patient 1

Systemic therapy was switched from oral voriconazole to oral posaconazole 300 mg twice daily. Two weeks after starting posaconazole, the exam showed no endoplaque, noting resolution of fungal infiltrate. However, the patient experienced hypertensive urgency, attributed to the posaconazole, which was discontinued immediately after a 4-week course. One month after discontinuation of oral posaconazole, clinical resolution of endoplaque and stromal infiltrate was noted (Fig. 4). Furthermore, lack of fungal elements on IVCM of the cornea was confirmed monthly for three months. Due to graft failure six months after, an optical keratoplasty was performed and the failed graft was confirmed by histopathology to be free of fungal elements.

#### Case 2

An 87-year-old female with a history of recurrent herpetic anterior uveitis of the left eye and bilateral primary open-angle glaucoma was referred to our department in March 2022 for concerns of microbial keratitis. The patient reported worsening pain and tearing in the left eye that was refractory to an antibiotic regimen of besifloxacin and erythromycin. On this initial visit, examination of the left eye revealed an inferotemporal corneal infiltrate with neovascularization and a best corrected visual acuity of 20/500. Like the previous patient, corneal scrapings obtained from the patient were taken for gram staining and culture examination using the standard microbiological culture techniques. The first corneal culture grew budding yeast with pseudohyphae. The patient was subsequently prescribed topical natamycin for fungal keratitis.

**Fig. 5:**
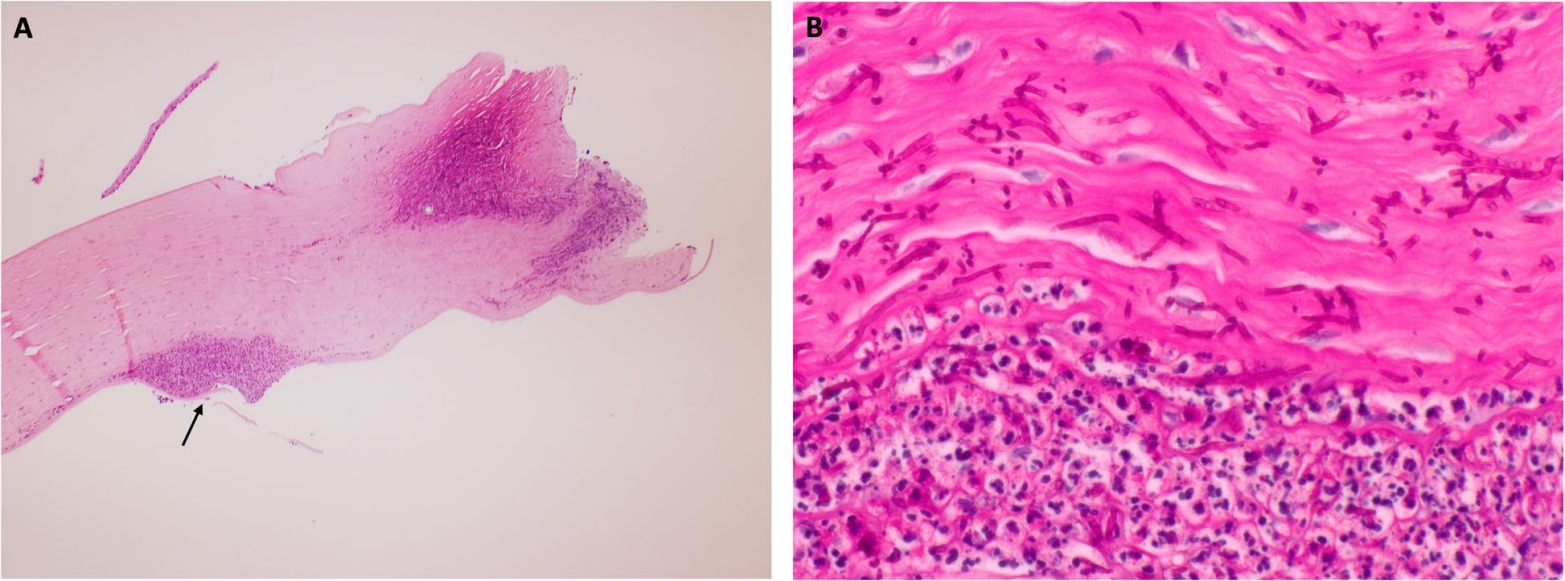
A Graft failure depicting acute necrotizing inflammation at the host-donor interface and disruption of Descemet membrane (arrow). H&E original magnification × 20. B. Deep stromal microabscesses containing numerous branching septate hyphae, highlighted with PAS-fungus, original magnification × 400

Ten days later, the culture was speciated as *Paecilomyces*. Broad-range fungal PCR identified *Paecilomyces ilacinum* species, the former name of *Purpureocillium lilacinum*, which belongs to the genus *Purpureocillium* [9]. Despite aggressive use of topical voriconazole and natamycin, there was progressive corneal thinning, worsening epithelial defect, and non-resolution of the stromal infiltrate. Therapeutic PKP of the left eye was performed with an inferiorly decentered trephination to include the entire infiltrate. Histopathology from the excised cornea was characterized by confluent microabscesses associated with hyphae-like fungal elements passing through Descemet membrane (Fig. 5).

**Fig. 6:**
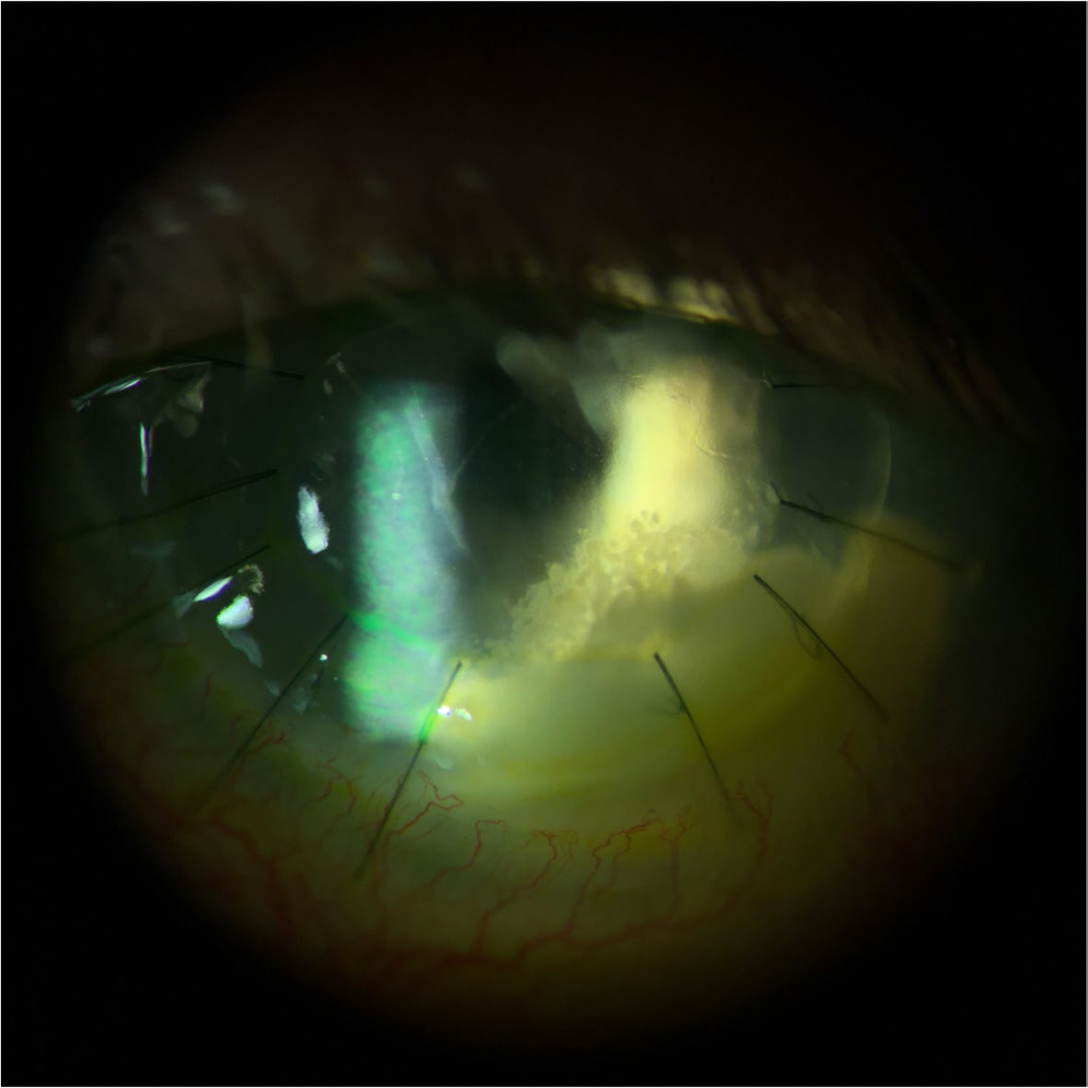
Progression of infiltrate at 6 o’clock graft-host junction

**Fig. 7:**
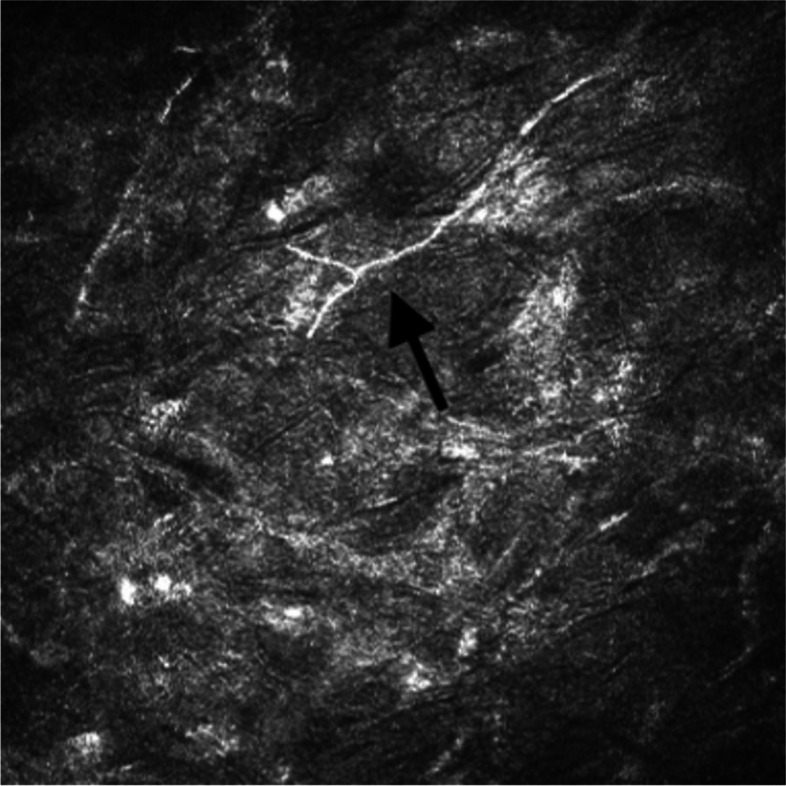
Long branching filaments demonstrate hyphae (arrow)

Topical antifungals were continued postoperatively in the absence of corticosteroids, but at the second postoperative week, there was a concern for intrastromal infiltrate surrounding the 6 o’clock suture. Intracameral and intrastromal voriconazole injections were administered. The suture was removed and sent for culture, which grew *Paecilomyces*, confirming recurrent infection. Progression of the infiltrate was noted at the sixth postoperative week (Fig. 6) and IVCM confirmed the presence of hyphae in the donor tissue (Fig. 7). Therefore, a decision was made to proceed to a second therapeutic PKP.

A repeat PKP of the left eye, as well as anterior chamber washout and administration of intracameral voriconazole was performed. At the first postoperative week, there was notable improvement in left eye visual acuity from counting fingers to 20/300. After the PKP, intracameral, intrastromal, and subconjunctival voriconazole injections were administered. Vision continued to improve in the following weeks, with examination of the left eye revealing a clear and fully epithelialized corneal graft. At the fourth postoperative week, a new infiltrate was observed in the deep stroma of the recipient rim associated with the 2–3 o’clock suture.

**Fig. 8:**
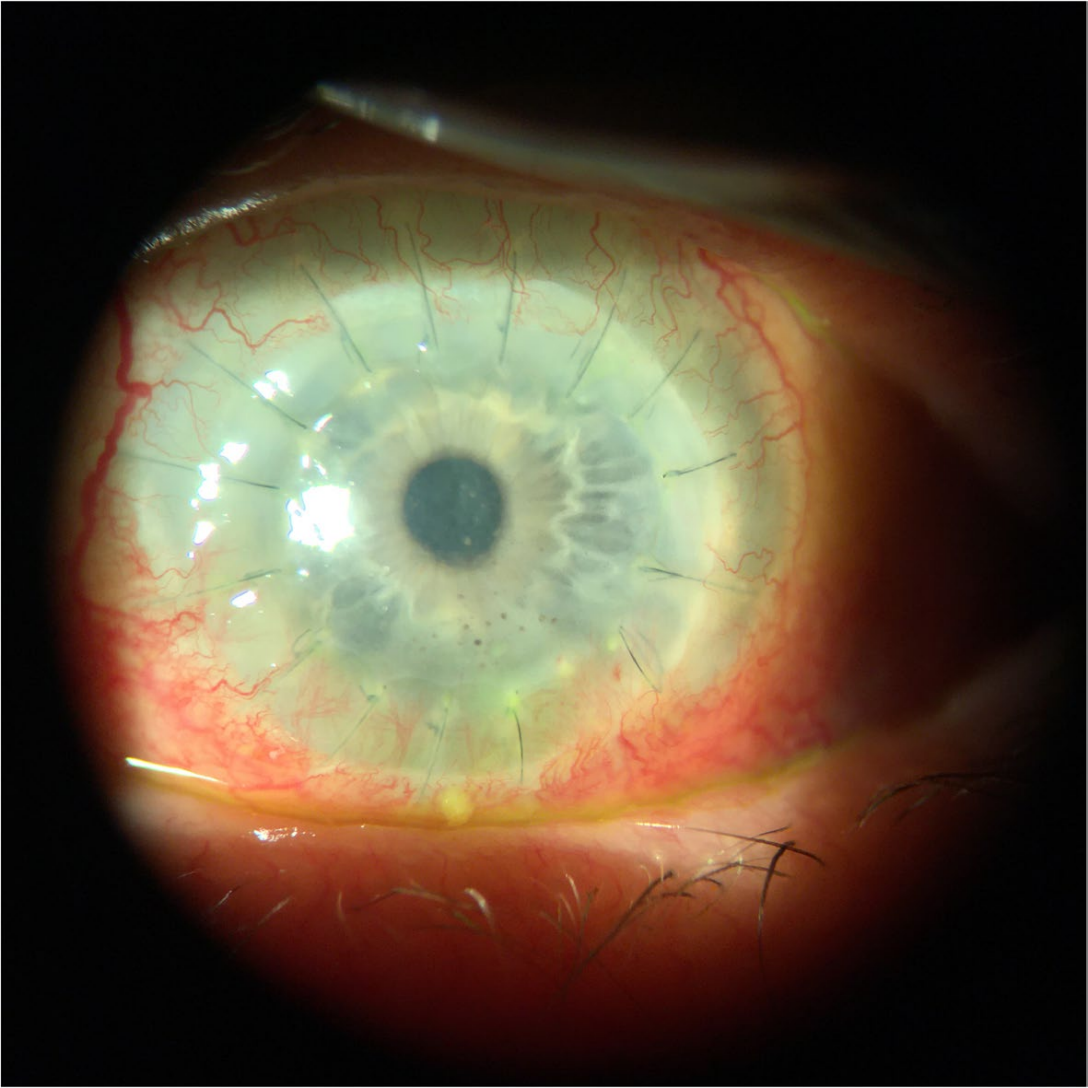
Resolution of infectious keratitis without recurrence following treatment with systemic Posaconazole for patient 2

Despite injections of amphotericin B and voriconazole given by intracameral, intrastromal, and subconjunctival routes, the infiltrate persisted. Systemic therapy was switched from voriconazole to posaconazole 300 mg twice daily. Due to concerns for hypertension and bleeding secondary to drug interactions between posaconazole and the patient’s previously prescribed apixaban, the posaconazole dose was halved. Nevertheless, due to uncontrolled hypertension, the patient was admitted to the medical intensive care unit, where posaconazole was discontinued as it was assumed to be the cause of her refractory hypertension. After three weeks of using posaconazole, the patient’s fungal keratitis appeared to have completely resolved (Fig. 8).

**Fig. 9:**
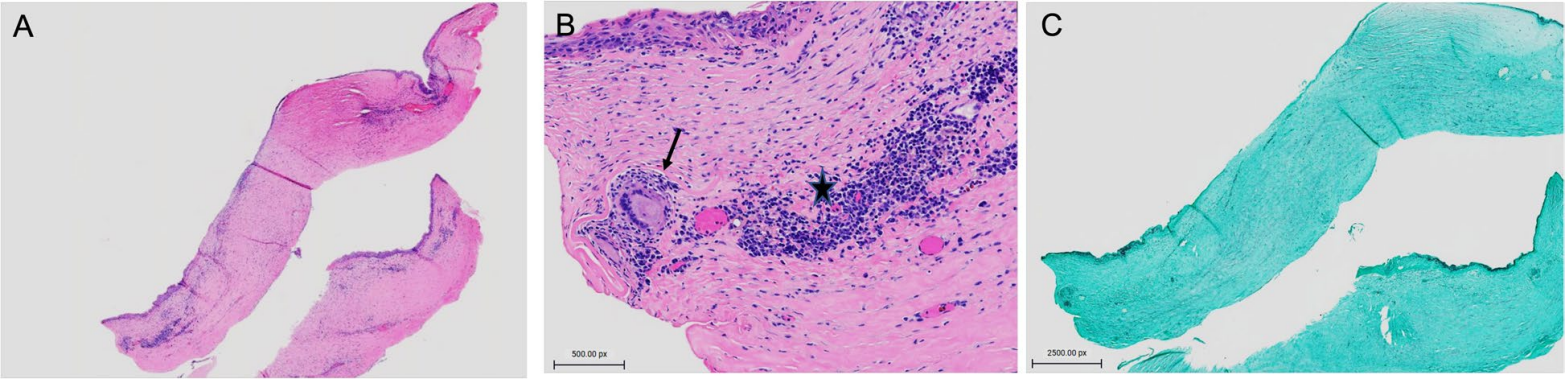
A Failed corneal graft with inflammatory pannus and stromal neovascularization. H&E × 10. B. Fragment of Bowman layer embedded in peripheral scar tissue with giant cell granulomatous reaction (arrow). Dense lymphoplasmacytic infiltrate surrounding new vessels in the stroma (asterisk). H&E × 100. C. Grocott methenamine silver stain failed to detect fungal elements. GMS × 20

Secondary graft failure was confirmed fifteen weeks after the second therapeutic PKP. An optical PKP was performed successfully, and the histopathology confirmed no evidence of fungal elements (Fig. 9).

#### Case 3

A 77-year-old diabetic male with a history of right PKP for prior corneal ulcer complicated by descemetocele formation was referred in April 2022 for evaluation of persistent epithelial defect on the donor cornea. He had glaucoma of both eyes, and his right eye visual acuity was hand motion. Despite previous tarsorrhaphy, self-retained amniotic membrane, and a trial of cenegermin, the epithelial defect had failed to close for almost one year. The patient was using topical moxifloxacin 0.5% and loteprednol 0.5% chronically during this time.

**Fig. 10:**
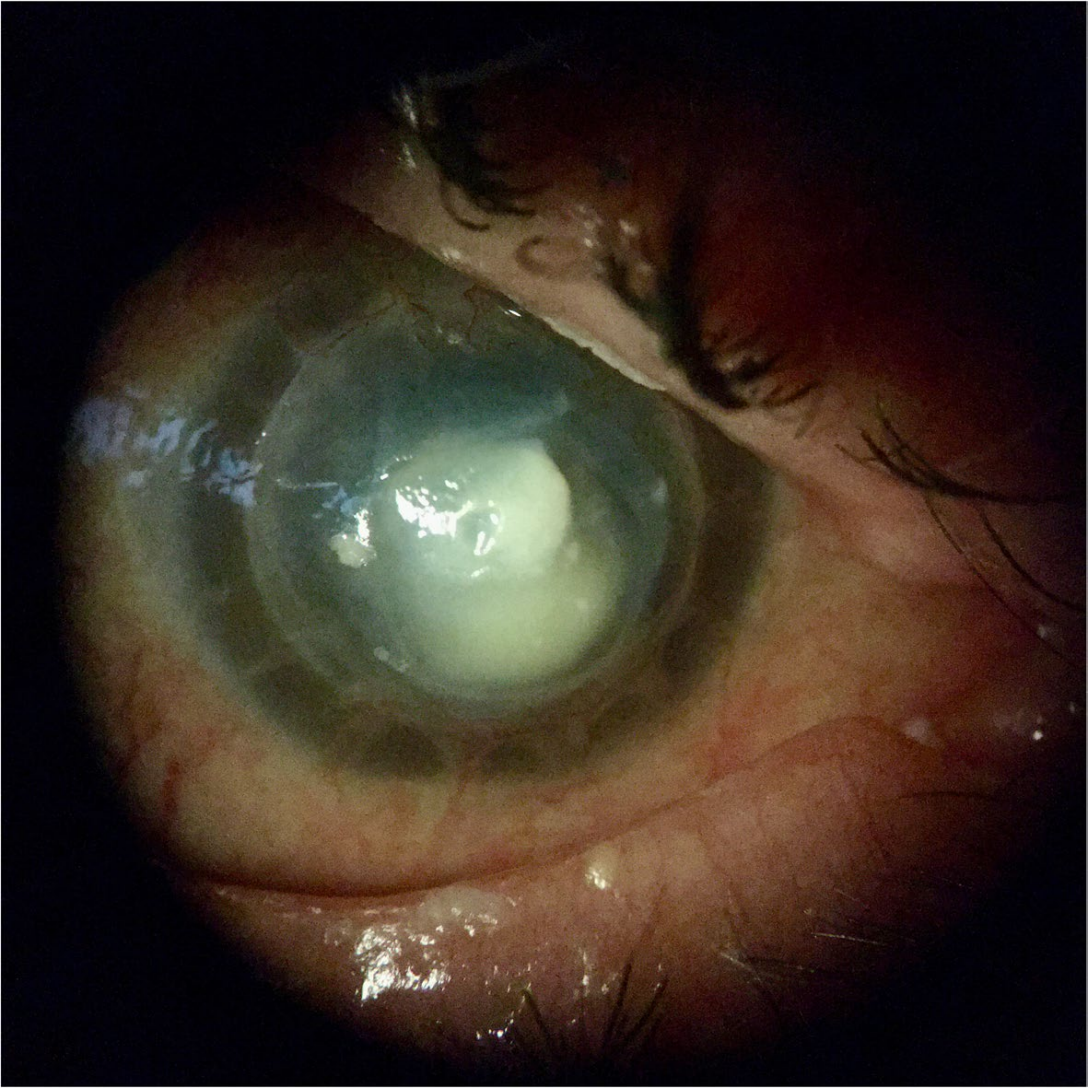
Elevated central stromal infiltrate with epithelial defect

**Fig. 11:**
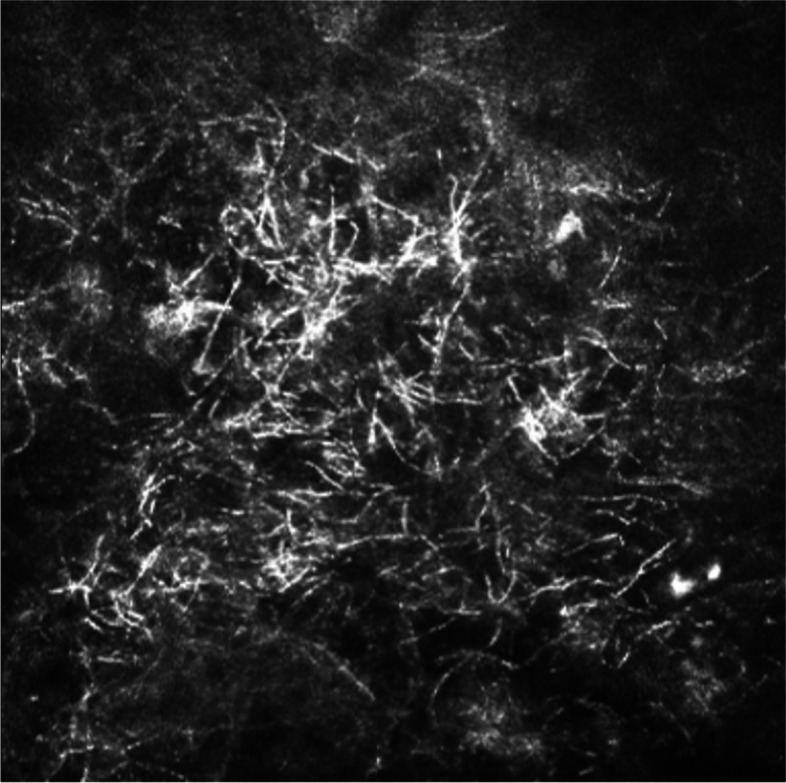
High density of hyphae is shown

**Fig. 12:**
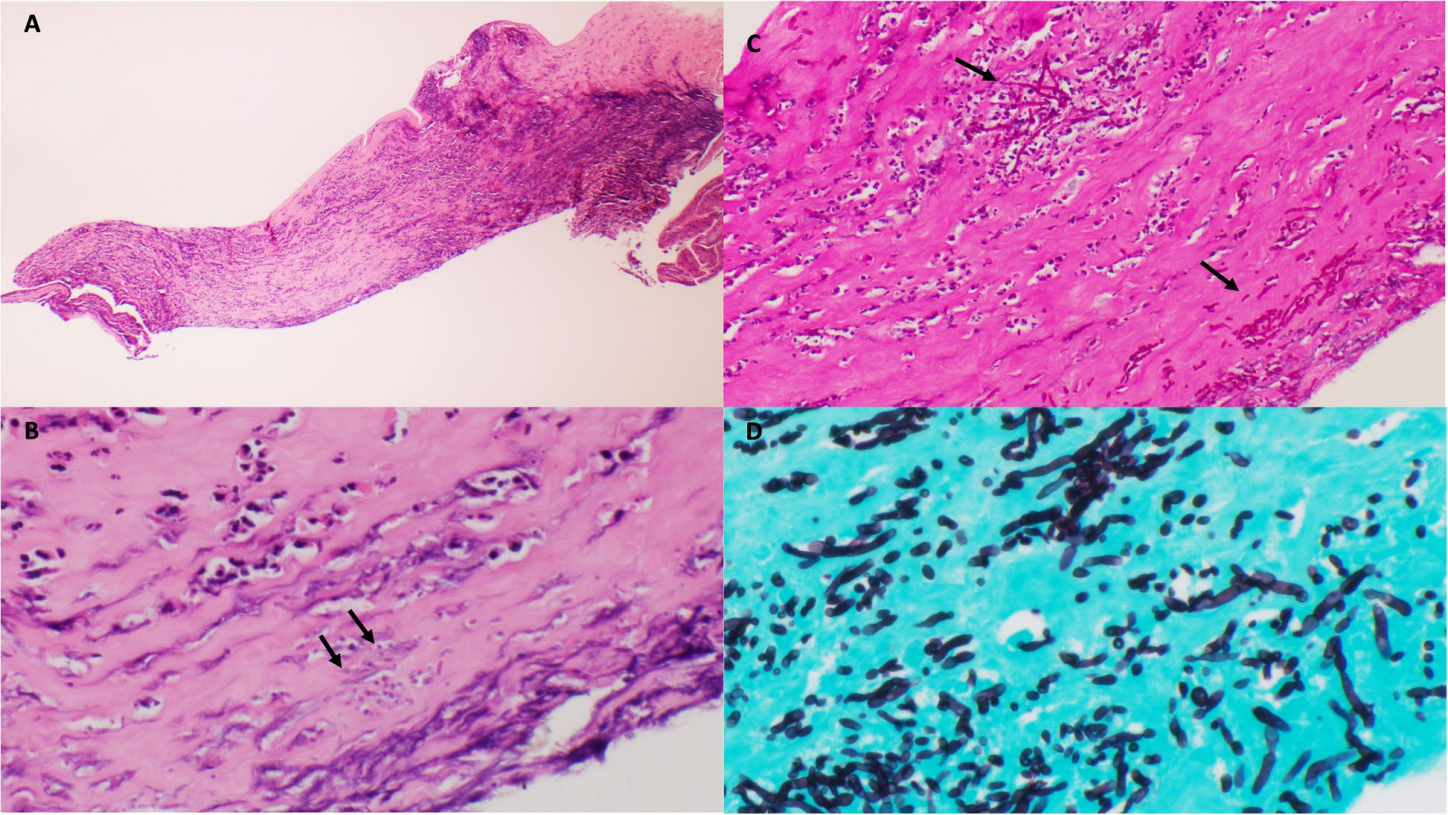
A Portion of cornea with disorganization of architecture and extensive stromal necrosis. H&E original magnification × 40. B. Neutrophils infiltrating stromal lamellae associated with fungal elements (arrows). H&E original magnification × 200. C. PAS stain highlighting hyphae in abscesses (arrows). Original magnification × 200. D. Branching septate hyphae and spores in the stroma. GMS stain, original magnification × 400

On exam, an infiltrate was noted in the bed of the epithelial defect on the donor cornea (Fig. 10). IVCM was performed over the infiltrate and showed the presence of hyphae (Fig. 11). A corneal perforation occurred within one week of starting topical natamycin and oral voriconazole, and a tectonic PKP was performed. Histopathologic evaluation of the excised cornea demonstrated numerous hyphae with focal septation, and yeast-like structures infiltrating necrotic stromal lamellae (best noted with PAS and GMS studies) (Fig. 12). Fungal culture grew *Paecilomyces*, identified through conventional morphologic methods. The patient began using natamycin, oral voriconazole, and moxifloxacin post-operatively without any topical steroids. Despite aggressive antifungal therapy, the cornea perforated again within one week, and a therapeutic PKP was performed. Post-operatively, the donor stroma proceeded to melt and, due to presumed poor prognosis, a Gundersen Flap and total tarsorrhaphy were performed to salvage the globe.

**Fig. 13:**
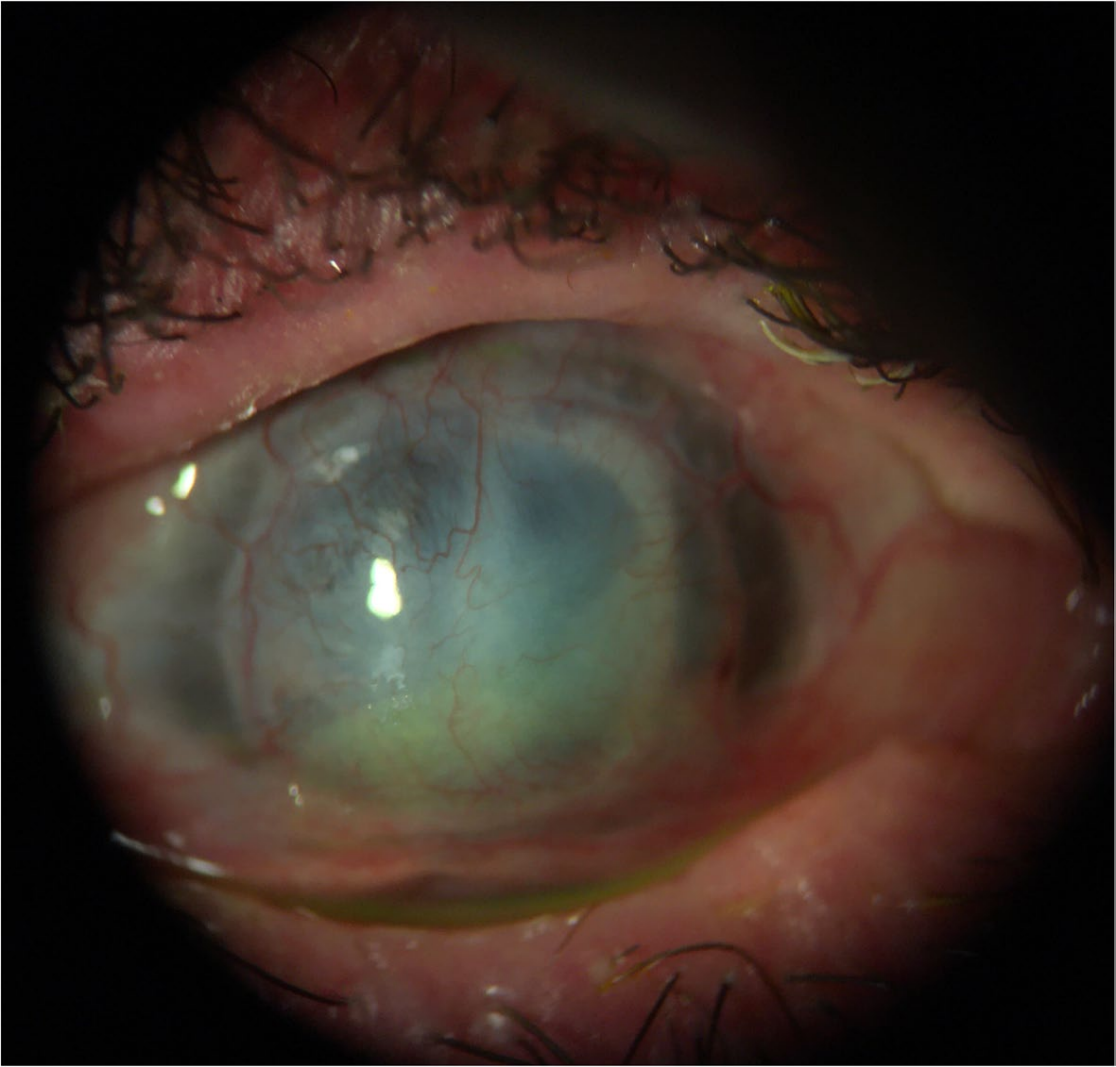
Resolution of infectious keratitis without recurrence following treatment with systemic Posaconazole for patient 3

Due to concern for residual fungal organisms and the difficulty of reliable penetration of topical medication given near total tarsorrhaphy, oral posaconazole 300 mg twice daily was initiated. The patient tolerated a three-week course of posaconazole, during which the ocular surface healed appropriately. The tarsorrhaphy was opened fourteen weeks after the Gunderson flap was performed (Fig. 13) and there has been no clinical evidence of recurrent microbial keratitis.

## Discussion

*Paecilomyces* is a rare but emerging cause of severe infections in humans, including keratitis [1]. Associated risk factors for *Paecilomyces* keratitis include previous ocular surgery, environmental trauma, and contact lens usage. Most cases of *Paecilomyces* keratitis are attributable to the *P. lilacinus* species [1]. The *P. lilacinus* species has become increasingly prevalent as a causal agent of infections and belongs to the genus *Purpureocillium* [9]*.*

*P. lilacinus* has been reported to transmit through the skin, indwelling catheters, or inhalation, with a tropic propensity for ocular structures. Interestingly, both in our report and the existing literature, contemporaneous *P. lilacinus* keratitis cases appear to present in geographically distinct clusters, suggesting that environmental factors play a role in pathogenesis [10]. Although the exact source of *P. lilacinus* has not been identified in our report, the observation that all three patients presented with the same infection during the same time of year suggests a shared etiology. Possible common sources, such as soil from gardening, water damage, and recent travel were explored, but no commonality was determined. Nevertheless, the patients shared characteristics that may have predisposed them to infection, including immunocompromised status, systemic disease, and usage of topical steroids. The clinical diagnosis of *P. lilacinus* infection is based on positive fungal culture and histopathology of corneal lesions. *P. lilacinus* can also be identified through its ability to sporulate in infected tissue [1, 10].

In the present study, IVCM was used in conjunction with histopathological assays and microbiologic testing to determine morphological changes in the cornea consistent with fungal keratitis. IVCM is a non-invasive imaging modality used for direct visualization of potential causative pathogens and associated cellular changes in real-time, on living organisms [11]. In patients with fungal keratitis, IVCM can be used to image corneal infiltrates with deep stromal involvement that are not accessible through scrapings alone. With a sensitivity and specificity of 67% and 100%, respectively, IVCM is a reliable and accurate way to diagnose fungal keratitis [12]. A prior study demonstrated a correlation between confocal and histopathologic findings in *Paecilomyces* spp. Keratitis [13]. Similarly, in our study, real-time confocal microscopy identified features of a filamentous fungus like spores and septate hyphae, corroborating positive fungal culture in all 3 cases. Furthermore, clinical resolution of infectious keratitis was supported by lack of confocal identification of fungal elements. This was further confirmed by histopathology in 2 of the 3 cases that underwent optical PKP after a long period of quiescence.

Timely management of fungal keratitis remains challenging. Negative or delayed culture results can impede accurate diagnosis [14]. Even with an accurate diagnosis, antifungal medications poorly penetrate the cornea. In the event of *P. lilacinus*-mediated keratitis, urgent medical intervention is needed to maintain corneal integrity and prevent the infection from spreading. In the discussed cases, the usage of steroids early in the treatment course may have negatively affected ocular outcomes. Our cases indicate that aggressive medical and surgical therapy is indicated given the recalcitrant nature of keratitis caused by this organism.

The Mycotic Ulcer Topical Treatment Trial I demonstrated that topical natamycin was more effective than topical voriconazole in preventing corneal perforation and therapeutic corneal transplants. It also showed significantly better visual acuity and ability to clear culture positivity after 6 days in patients receiving topical natamycin [15]. Unfortunately*, P. lilacinus* demonstrates resistance to conventional antifungal medication and patients often experience poor outcomes [1] despite aggressive medical and surgical intervention. Several reports have noted high-dose oral posaconazole as an effective medical option for fungal keratitis recalcitrant to first-line antifungals [16–18]. Although posaconazole has shown significant improvement on a case-by-case basis, it can cause serious side effects, including gastrointestinal complaints, headaches [18], elevated liver function tests [19], and rarely hepatotoxicity and cardiotoxicity [20]. All three patients in the present study were initially prescribed oral voriconazole, then switched to Posaconazole 600 mg for 2 weeks. The dose was lowered to 300 mg after improvement was noted. Treatment with high-dose oral posaconazole led to clinical resolution of infection that was not achieved with oral voriconazole combined with topical and local injections of antifungals. However, two of our patients experienced severe hypertension, which did not resolve with reduced dosing, prompting the discontinuation of posaconazole.

## Conclusion

We have reported three cases of recalcitrant *Paecilomyces* keratitis presenting to the same institution within a short period. Recognizing the potential for corneal *Paecilomyces* infection and adverse systemic side effects in response to antifungal therapy is crucial to ensure prompt medical attention and appropriate treatment for these patients. Guidelines discussing the optimal treatment for primary *Paecilomyces* corneal infections should include points regarding the management of therapy-related side effects. Ophthalmologists should be instructed to recognize the signs and symptoms of fungal infections and associated therapies. In our series, the microbiologic diagnosis of fungal organism as a cause of infectious keratitis was corroborated by in vivo histology obtained by IVCM imaging and confirmed with histopathologic findings from the excised corneal buttons.

## Data Availability

Not applicable.

## Abbreviations

GMS: Grocott’s methenamine silver
IVCM: In vivo confocal microscopy
PAS: Periodic acid-schiff
PKP: Penetrating Keratoplasty
